# The evolution of arch surgery: Frozen elephant trunk or conventional elephant trunk?

**DOI:** 10.3389/fcvm.2022.999314

**Published:** 2022-10-20

**Authors:** Amalia I. Moula, Jamie L. R. Romeo, Gianmarco Parise, Orlando Parise, Jos G. Maessen, Ehsan Natour, Elham Bidar, Sandro Gelsomino

**Affiliations:** Cardiovascular Center, CARIM School for Cardiovascular Disease, University Hospital Maastricht, University of Maastricht, Maastricht, Netherlands

**Keywords:** elephant trunk, frozen elephant trunk, antegrade stent, aortic surgery, aortic aneurysm

## Abstract

Treatment of aortic arch aneurysms and dissections require highly complex surgical procedures with devastating complications and mortality rates. Currently, repair of the complete arch until the proximal descending thoracic aorta consists of a two-stage procedure, called elephant trunk (ET) technique, or a single stage a single-stage technique referred to as frozen elephant trunk (FET). There is conflicting evidence about the perioperative results of ET in comparison with FET. We carried out a meta-analysis to investigate possible differences in perioperative and early (up to 30 days) outcomes of ET vs. FET, particularly for mortality, spinal cord injury (SCI), stroke, and renal failure. We also performed a meta-regression to explore the effects of age and sex as possible cofactors. Twenty-one studies containing data from interventions conducted between 1997 and 2019 and published between 2008 and 2021 with 3153 patients (68.5% male) were included. ET was applied to 1,693 patients (53.7%) and FET to 1460 (46.3%). Overall mortality after ET was 250/1693 (14.8%) and after FET 116/1460 (7.9%). Relative risk (RR) and 95% confidence interval (CI) were 1.37 [1.04 to 1.81], *p* = 0.027. There was no significant effect of age and sex. SCI occurrence after the second stage of ET was 45/1693 (2.7%) and after FET 70/1,460 patients (4.8%) RR 0.53 [0.35 to 0.81], *p* = 0.004. Age and sex were not associated with the risk of SCI. No significant differences were observed between ET and FET in the incidence of stroke and renal failure. Our results indicate that ET is associated with higher early mortality but lower incidence of SCI compared to FET. When studies published in the last 5 years were analyzed, no significant differences in mortality or SCI were found between ET and FET. This difference is attributed to a decrease in mortality after ET, as the mortality after FET did not change significantly over time.

## Introduction

The incidence of aortic aneurysms and dissections is on the rise during the last two decades and can affect between 2 and 6 out of 100,000 people every year (1–4).

Aortic arch dissections arise from intimal tears within an aneurysm and are highly deadly with a mortality rate reaching up to 80% if there is a rupture. The risk of dying is around 21% for patients who reach the hospital alive (5, 6).

With increasing incidence and high mortality, there is a need for better management and treatment of both aortic aneurysms and dissections.

Aneurysms are permanent dilatations of the aorta with at least 50% increase of the expected normal diameter of the aorta. Dissections are highly fatal conditions in which disruptions (tears) of the aortic intimal layer cause rushing of blood within the vessel wall, forcing the layers to dissect (7). Aneurysms can occur without dissections and vice versa (7, 8). Current guidelines indicate that “total aortic arch replacement with or without an elephant trunk extension is indicated when a tear within the arch extends into the proximal descending aorta or extends throughout the arch on the greater curve’s aspect, i.e., involves separation or disruption of the Ostia of the brachiocephalic vessels, precluding the ability to reconstruct a neomedia effectively” (9).

Treatment of both dissections and aneurysms in the aortic arch can be done with a two-stage surgical technique called “elephant trunk” (ET) (10). The first stage of ET involves a sternotomy and a reconstruction of the ascending aorta and the aortic arch. At the second stage, a floating extension (elephant trunk) in the descending aorta is used in order to extend the repair toward descending thoracic aorta. An important limitation of this technique is the fact that almost half of the patients that undergo the first step, never arrive at the second step, usually because they die or because they refuse a second operation (11).

The evolution of surgical and endovascular techniques has resulted in the development of a composite prosthesis, known as the “Frozen elephant trunk” (FET) (9, 11). In this technique a stent graft is implanted in the proximal descending thoracic aorta through the aortic arch prosthesis, during the primary procedure (9).

The use of FET instead of the conventional ET is increasing in the last years. However, there is contrasting evidence about which intervention has less complications and whether a real difference exists in their outcomes (12, 13).

Our aim in this meta-analysis was to assess the outcomes of the two techniques for Debakey type I or Stanford type A aortic dissections. We also analyzed separately the data from papers published during the last 5 years (2017–2021) in order to detect possible different outcomes, as newer devices are being developed and techniques evolved (14).

## Materials and methods

### Search strategy

The literature search was performed in accordance with the principles of the Cochrane Handbook (8) and the Preferred Reporting Items for Systematic Reviews and Meta-Analyses (PRISMA) (15, 16).

An unrestricted literature search was performed in PubMed, Web of Science, Scopus, and the Cochrane library.

The search aimed at finding articles reporting outcomes in patients undergoing aortic arch surgery with ET or FET and contained separate relevant data for each type of intervention.

A systematic search was conducted in PubMed combining MeSH and free terms for the clinical situations, the treatments, and the outcomes. An additional search was conducted in the references of the examined articles.

The search strategy was designed by two authors (AM and JR), and their decisions were approved by a third author (SG). The literature search was performed by one author (AM). The eligibility of the selected articles eligibility and the risk of bias were assessed independently by two independent reviewers (EN, EB). The Risk of Bias in Non-randomized Studies-of Interventions (ROB-INS-I) tool (17) was used by two reviewers (GP and OP) for the assessment of bias of each study. A third reviewer (JGM) resolved any possible differences in the assessment.

The following domains were examined in each study for the risk of bias, according to the Cochrane Handbook: (1) in the confounding; (2) in the participant selection of each study; (3) in the classification of interventions; (4) in cases of deviations from intended interventions; (5) in cases of missing data; (6) in the estimation of outcomes; (7) in the selection of the result to be reported; and (8) overall assessment of bias. The Risk-of-Bias VISualization (robvis) software was used for producing the plot for ROBINS-I (17).

### Selection process

The following inclusion criteria were used for the selection of articles: (1) human studies; (2) full research articles containing data on the outcomes of both ET and FET and (3) studies including at least 10 patients.

The exclusion criteria for the article selection were: (1) non-human studies, (2) case reports, (3) previous reviews and/or meta-analyses, (4) editorials, and (5) studies without adequate demographic and/or outcome information on both ET and FET interventions, and (6) studies in languages other than English.

### Quality assessment

A rating scale based on the Downs and Black checklist for measuring was used for assessing the quality of included studies (18). The assessment was done using a version with 18 items. The items were ranked with the use of a binary score (0 or 1) except for two items that were rated on a scale from 0 to 2 and from 0 to 5, respectively.

The ratings were collected by two independent researchers (AIM and JLRR). A third reviewer (OP) resolved divergences and quantified the ratings with the use of Cohen’s kappa (19).

### Statistical analysis

The R software v. 3.6.1 (R Foundation for Statistical Computing, Vienna, Austria) was used for conducting the meta-analysis. The relative risk (RR) was used as index statistics. The results of RR are presented with two decimal points followed by the 95% confidence interval (CI) in brackets in the form: RR (lower CI, upper CI). As heterogeneity among studies was expected, the random effects model was used. The statistical inconsistency Higgin’s *I*^2^ test was used for the evaluation of heterogeneity (20). *I*^2^ values > 75% were considered to have high heterogeneity and *I*^2^ values < 40% were considered to have low heterogeneity. The Egger’s test of the intercept was used for assessing publication bias. A meta-regression analysis was conducted in order to assess the impact of the potential interaction factors, such as age and sex *p*-values < 0.05 were considered statistically significant.

### Endpoints

The primary endpoints of this study were: early mortality, defined as the occurrence of mortality during hospitalization or up to 30 days after the intervention, spinal cord injury/ischemia (SCI) and/or paraplegia and/or quadriplegia, stroke, and renal failure. The possible impact of age and sex on the above-mentioned parameters was also examined.

## Results

### Search results and characteristics of the studies

**FIGURE 1:**
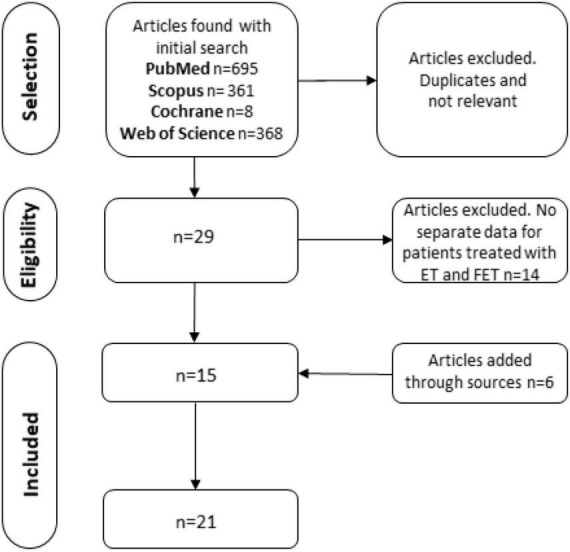
Preferred reporting items for systematic reviews and meta-analyses (PRISMA) flow chart of the article selection process. ET, elephant trunk; FET, frozen elephant trunk.

The initial search resulted in 695 results from PubMed, 368 from Web of Science, 361 from Scopus, and 8 from the Cochrane library. After the elimination of duplicates and the application of the inclusion and exclusion criteria, 29 articles containing data on ET and FET were found. The full text of these articles was then examined. Fourteen articles did not contain separate data for patients treated with ET and FET and were excluded. An additional search in the references of the selected articles resulted in 6 additional articles that were also included (Figure 1). Overall, twenty-one studies were included in the analysis.

The 21 selected studies (21–41) included a total of 3153 patients. The studies contained data from interventions conducted between 1997 and 2019 and were published between 2008 and 2021. The ET was applied in 1693 patients (53.7%) and FET in 1460 (46.3%). In 17 out of the 18 studies that contained detailed data about sex, 2,105 of 3,075 patients (68.5%) were male. One of the selected studies (38) contained two different cohorts, one with acute and one with chronic dissections. These cohorts were included as separate. One article contained two overlapping cohorts, one cohort with all the patients examined and one with propensity-matched patients (33). Only the matched cohort was included in the statistical evaluation.

The studies that were included in this meta-analysis are shown in Table 1. The patients’ characteristics are shown in Table 2.

**TABLE 1:** Studies included in the meta-analysis.

Study #	References	Clinical situation (as reported)	Study type	Centers	Period	Total patients (*n*)
1	Jakob et al. (27)	Acute DeBakey type I dissection	Retrospective observational	1	2001–2007	45
2	Pochettino et al. (34)	Acute DeBakey I dissection	Retrospective observational	1	2005–2008	78
3	Uchida et al. (39)	Acute type A aortic dissection	Retrospective observational	1	1997–2008	120
4	Sun et al. (38) acute	Type A aortic dissection acute	Retrospective observational	1	2003–2008	214
5	Sun et al. (38) chronic	Type A aortic dissection chronic	Retrospective observational	1	2003–2008	197
6	Hofferberth et al. (25)	DeBakey type I dissection	Retrospective observational	1	2003–2011	37
7	Leontyev et al. (29)	Total aortic arch replacement	Retrospective observational	1	2003–2011	171
8	Di Eusanio et al. (22)	Extensive aortic aneurysm	Retrospective observational	2	2003–2011	57
9	Preventza eyt al. (35)	Acute type I aortic dissection	Retrospective observational	1	2005–2012	112
10	Vallabhajosyula et al. (40)	Acute DeBakey type I aortic dissection	Retrospective observational	1	2005–2012	242
11	Shrestha et al. (37)	Total aortic arch replacement	Retrospective observational	1	2001–2013	277
12	Matt et al. (30)	acute type A aortic dissection	Prospective observational	1	2010–2016	74
13	Preventza et al. (36)	Chronic dissecting and atherosclerotic aneurysms	Retrospective observational	1	2010–2015	129
14	Alhussaini et al. (21)	Aortic arch repair	Retrospective observational	1	2003–2016	118
15	Furutachi et al. (23)	Type A acute aortic dissection	Retrospective observational	1	2010–2018	50
16	Inoue et al. (26)	Type A acute aortic dissection	Retrospective observational	1	2012–2018	148
17	Mkalaluh (31)	Total arch replacement	Retrospective observational	1	2001–2017	50
18	Mutsuga et al. (32)	Total arch replacement	Retrospective observational	1	1997–2015	91
19	Hage et al. (24)	Aortic arch repair	Retrospective observational	9	2002–2018	390
20	Ogino et al. (33)	Aortic arch repair	Retrospective observational	41	2016–2019	388
21	Vendramin et al. (41)	Aortic arch replacement	Retrospective observational	1	2017–2021	39
22	Koizumi et al. (28)	Total aortic arch replacement	Retrospective observational	1	2011–2019	126

**TABLE 2:** Included studies and patients’ characteristics.

		Conventional elephant trunk (ET)							Frozen elephant trunk (FET)						
Study #	References	Patients (*n*)	Male	Age	In-hospital/30 day mortality	SCI	Stroke	RF	Patients (*n*)	Male	Age	In-hospital/30 day mortality	SCI	Stroke	RF
1	Jakob et al. (27)	23	18	55 ± 15	5	0	2	11	22	17	57 ± 12	2	0	2	12
2	Pochettino et al. (34)	42		61 ± 13	6	1	4	7	36		59 ± 13	5	3	1	6
3	Uchida et al. (39)	55	25	72.3	2	0	0	1	65	28	64.4	3	0	0	3
4	Sun et al. (38) acute	66	36	46 ± 13	4	1	1	2	148	126	45 ± 11	7	3	4	1
5	Sun et al. (38) chronic	54	36	45 ± 14	2	0	0	0	143	112	45 ± 10	2	4	3	2
6	Hofferberth et al. (25)	18	13	59 ± 13	2	1	3	6	19	16	54 ± 12	1	0	2	5
7	Leontyev et al. (29)	125	80	61 ± 13	51	5	20	23	46	23	69 ± 10	8	10	6	11
8	Di Eusanio et al. (22)	36	19	64.3 ± 10.8	5	4	2	2	21	18	65.6 ± 7.3	1	3	2	2
9	Preventza et al. (35)	87	63	57 (48–66)	25	1	9	10	25	22	64 (48–73)	6	2	3	4
10	Vallabhajosyula et al. (40)	180	125	59.4 ± 13.9	25	4	15	42	62	40	58.2 ± 11.9	6	4	3	13
11	Shrestha et al. (37)	97	59	59.7 ± 12.7	24	5	12	12	180	126	59.8 ± 13.2	22	9	24	25
12	Matt et al. (30)	37	26	60 (± 12)	5	1	9		37	22	60 (± 15)	0	0	3	
13	Preventza et al. (36)	92	68	64.0 (53.5–69.5)	21	3	5	0	37	21	68.0 (64–73)	15	2	2	2
14	Alhussaini et al. (21)	70	42	65.67 ± 13.3	9	4	10		48	31	64 ± 11	8	2	3	
15	Furutachi et al. (23)	30	17	58.5 ± 12.5	3	2	2		20	15	58.8 ± 9.4	1	0	0	
16	Inoue et al. (26)	115	74	67 ± 11	16	0			33	19	0	4	0		
17	Mkalaluh (31)	25	15	66 [58–76]	8	4	2	6	25	14	69 [60–72]	5	1	6	6
18	Mutsuga et al. (32)	37	32	68.5 ± 9.6	0	2	3	1	54	41	68.5 ± 9.6	2	9	5	4
19	Hage et al. (24)	218	138	63 ± 13	28	4	27		172	120	65 ± 13	15	9	22	
20	Ogino et al. (33)	194	144	68.4 ± 12.5	0	1	15	9	194	137	68.9 ± 10.7	2	6	16	11
21	Vendramin et al. (41)	26	15	66 ± 2	1	2	5	18	13	8	55 ± 9	0	1	1	5
22	Koizumi et al (28)	66	53	74.1 ± 9.4	8	0	2	8	60	51	76.2 ± 5.9	1	2	4	3

SCI, spinal cord injury including paraplegia/quadriplegia; RF, renal failure.

Of the 21 selected studies, 10 were published until 2016 and 11 were published in the last 5 years between 2017 and 2021. The studies published until 2016 included data on interventions conducted from 1997 to 2013. The 11 most recent studies published in 2017 and after, included data on more recent interventions ranging until 2021. A total of 1,603 patients, of which 1103 (72.3%) were male were included in these studies. ET was applied in 910 patients (56.8%) and FET in 693 (43.2%).

### Quality of the studies

The average quality rating of all the studies was 0.83 ± 0.08. The quality assessment is shown in Supplementary Table 1 in the supplement. The studies were rated with a median rating of 0.83 [IQR 0.78 to 0.89], ranging from 0.67 to 1.00.

### Elephant trunk vs. frozen elephant trunk

The RR of ET vs. FET, the *I*^2^ values and respective *p*-values, the Egger’s test results, the funnel plot asymmetry tests *p*-values, and the *p*-values of the meta-regression for the effects of age and sex are presented in Table 3. T he results for all the examined papers published between 2008 and 2021 and for those published between 2017 and 2021 are presented in separate lines in the table.

**TABLE 3:** Elephant trunk (ET) vs frozen elephant trunk (FET) odds ratios and 95% confidence intervals (CI) and probability (p) of effects of age and sex (% male patients).

Parameter	Articles published in years:	ET vs. FET relative risk [95% CI]	*p*	*I*^2^ value and p	Egger’s test: x-intercept [95% CI]	Funnel plot asymmetry test p	Effect of age	Effect of sex (% male)
Mortality	2008–2021	1.37 [1.04–1.81]	***p* = 0.027**	27.71%, *p* = 0.19	0.15 [−0.50 to 0.80]	*p* = 0.50	*p* = 0.435	*p* = 0.804
	2017–2021	1.11 [0.71–1.74]	*p* = 0.646	32.38%, *p* = 0.11	−0.29 [−1.65 to 1.06]	0.34	*p* = 0.785	*p* = 0.968
SCI	2008–2021	0.53 [0.35–0.81]	***p* = 0.004**	11.93%, *p* = 0.53	−1.23 [−2.55 to 0.09]	*p* = 0.21	*p* = 0.612	*p* = 0.275
	2017–2021	0.61 [0.34–1.12]	*p* = 0.113	3.85%, *p* = 0.43	−1.36 [−3.18 to 0.45]	*p* = 0.33	*p* = 0.131	***p* = 0.029**
Stroke	2008–2021	1.06 [0.83–1.37]	*p* = 0.639	0.0%, *p* = 0.89	−0.03 [−0.38 to 0.32]	*p* = 0.64	*p* = 0.508	*p* = 0.349
	2017–2021	1.06 [0.76–1.47]	*p* = 0.732	0.0%, *p* = 0.43	−0.14 [−0.96 to 0.68]	*p* = 0.56	***p* = 0.044**	*p* = 0.326
Renal failure	2008–2021	0.98 [0.79–1.23]	*p* = 0.892	0.0%, *p* = 0.72	0.17 [−0.69 to 1.02]	*p* = 0.37	*p* = 0.947	*p* = 0.896
	2017–2021	1.15 [0.71–1.88]	*p* = 0.569	12.93%, *p* = 0.19	1.11 [4.78 to −2.56]	*p* = 0.15	*p* = 0.803	*p* = 0.813

SCI, spinal cord injury. Results for all the examined articles published. Statistically significant probabilities (p < 0.05) are in bold.

**FIGURE 2:**
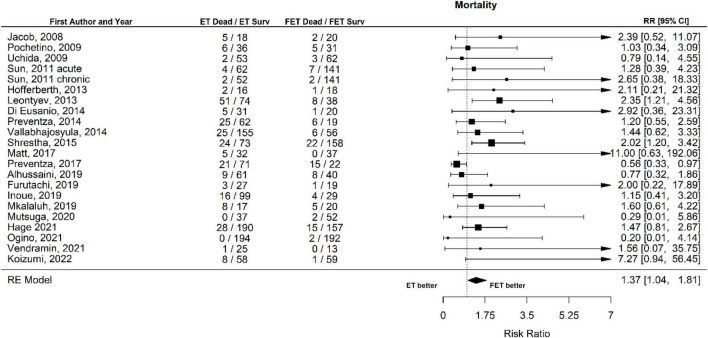
Relative risk of mortality in patients undergoing aortic interventions with conventional elephant trunk (ET) vs. frozen elephant trunk (FET). Forest plot. ET vs. FET *p* = 0.027.

**FIGURE 3:**
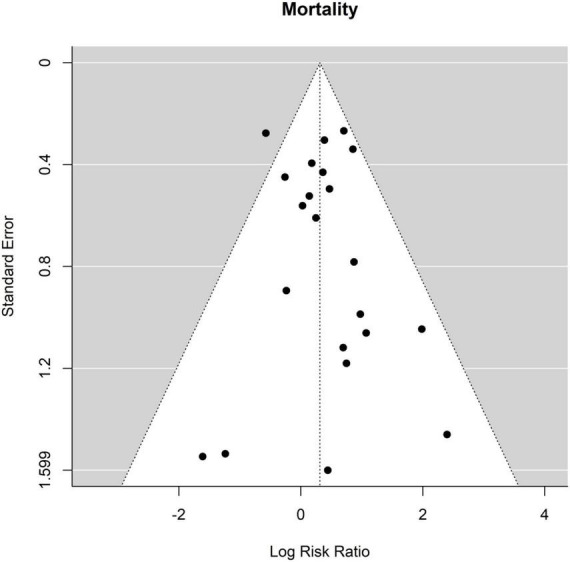
Relative risk of mortality in patients undergoing aortic interventions with conventional elephant trunk (ET) vs. frozen elephant trunk (FET). Funnel plot.

The overall mortality after ET was 250 of 1,693 patients (14.8%) and after FET 116 of 1,460 patients (7.9%). The relative risk of mortality after ET vs. after FET was 1.37 [1.04 to 1.81], (*p* = 0.027, *I*^2^ = 27.71%, *p*-value *I*^2^ = 0.19; Egger’s test 0.15 [−0.50 to 0.80], indicating a trend for higher mortality risk with conventional ET (Figure 2, Forest plot). The funnel plot is shown in Figure 3 (asymmetry test: *p* = 0.50). There was no significant effect of age and sex (percentage of male patients) on mortality according to the meta-regression analysis (Table 3).

**FIGURE 4:**
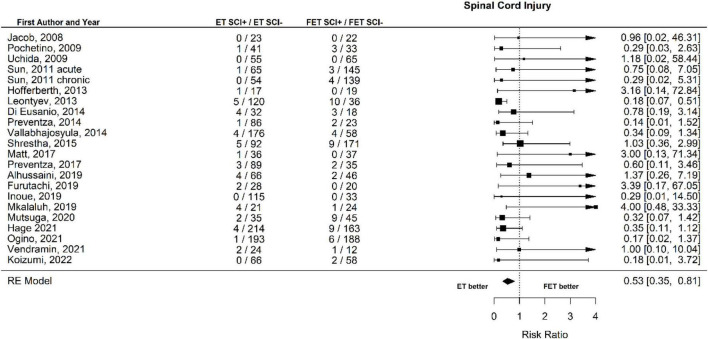
Relative risk of spinal cord injury (SCI) and hemiplegia/paraplegia in patients undergoing aortic interventions with conventional elephant trunk (ET) vs. frozen elephant trunk (FET). Forest plot. ET vs. FET: *p* = 0.004.

**FIGURE 5:**
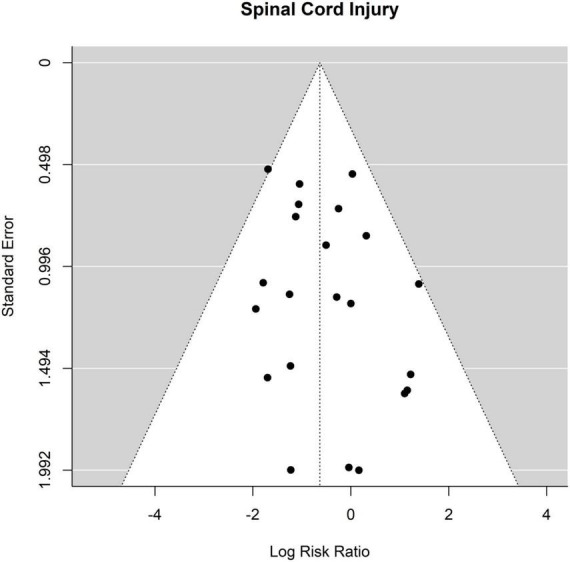
Relative risk of spinal cord injury (SCI) and hemiplegia/paraplegia in patients undergoing aortic interventions with conventional elephant trunk (ET) vs. frozen elephant trunk (FET). Funnel plot.

The occurrence of SCI after ET was 45 out of 1693 patients (2.7%) and after FET was 70 out of 1,460 patients (4.8%). The RR for SCI of ET vs. FET was found to be 0.53 [0.35 to 0.81], (*p* = 0.004, *I*^2^ = 11.93%, *p*-value *I*^2^ = 0.53; Egger’s test −1.23 [−2.55 to 0.09]. FET was associated with a significantly higher risk for SCI (Figure 4, Forest plot). The funnel plot is shown in Figure 5 (asymmetry test: *p* = 0.21). Age and sex were not associated with the risk of SCI (Table 3).

**FIGURE 6:**
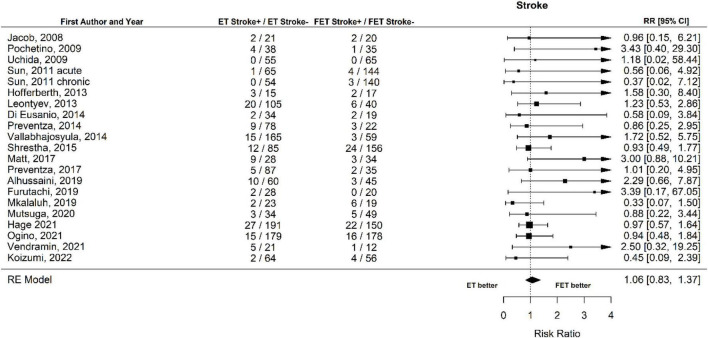
Relative risk of stroke in patients undergoing aortic interventions with conventional elephant trunk (ET) vs. frozen elephant trunk (FET). Forest plot *p* = 0.89.

**FIGURE 7:**
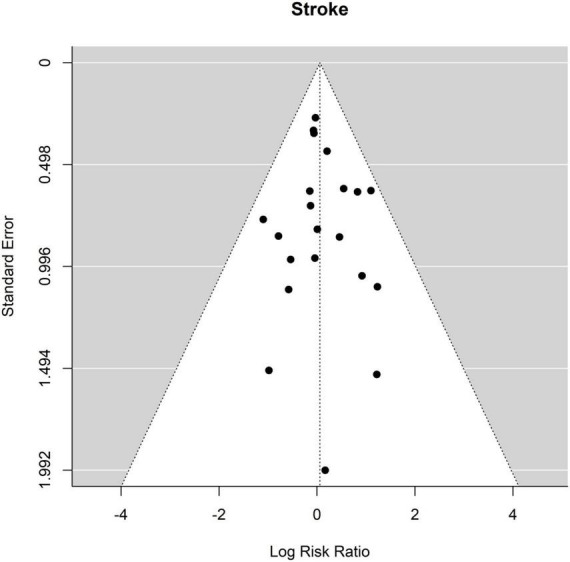
Relative risk of stroke in patients undergoing aortic interventions with conventional elephant trunk (ET) vs. frozen elephant trunk (FET). Funnel plot.

Twenty out of the twenty-one studies contained sufficient data on the occurrence of stroke. No significant differences were found between ET and FET in the risk of stroke. The occurrence of stroke was 9.4% (148 of 1,578 patients) after ET and 7.8% (112 of 1,427 patients) (Figure 6, Forest plot). The RR was 1.06 [0.83 to 1.37] (*p* = 0.64, *I*^2^ = 0.0%, *p*-value *I*^2^ = 0.89; Egger’s test −0.03 [−0.38 to 0.32]. The funnel plot is shown in Figure 7 (asymmetry test: *p* = 0.64). There was no effect of age or sex on the outcome of stroke (Table 3).

**FIGURE 8:**
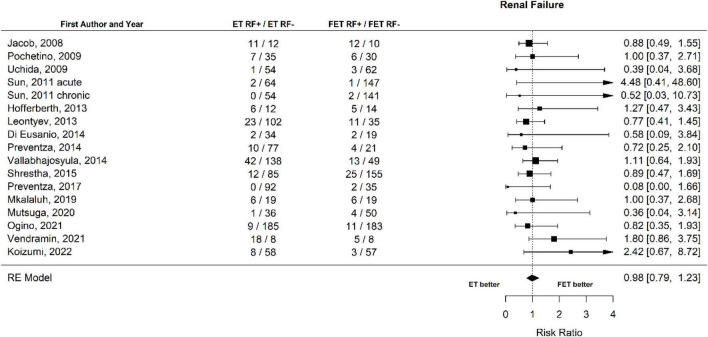
Relative risk of renal failure (RF) in patients undergoing aortic interventions with conventional elephant trunk (ET) vs. frozen elephant trunk (FET). Forest plot *p* = 0.72.

**FIGURE 9:**
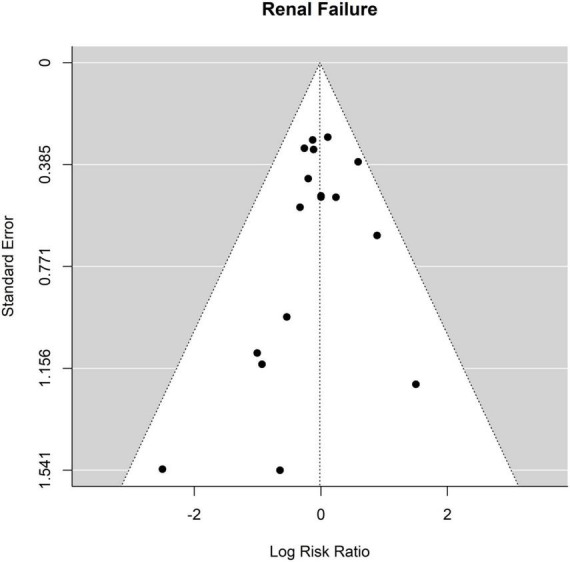
Relative risk of renal failure (RF) in patients undergoing aortic interventions with conventional elephant trunk (ET) vs. frozen elephant trunk (FET). Funnel plot.

Sixteen of the twenty-one studies contained data about renal failure. The occurrence of renal failure was 12.9% (158 of 1,223 patients) after ET and 10.0% (115 of 1,150 patients) (Figure 8 Forest plot). The RR of ET vs. FET was 0.98 [0.79 to 1.23] (*p* = 0.89, *I*^2^ = 0.0%, *p*-value *I*^2^ = 0.72; Egger’s test 0.17 [−0.69 to 1.02]. The funnel plot is shown in Figure 9 (asymmetry test: *p* = 0.37). There was no significant effect of age or sex on the outcome of renal failure (Table 3).

**FIGURE 10:**
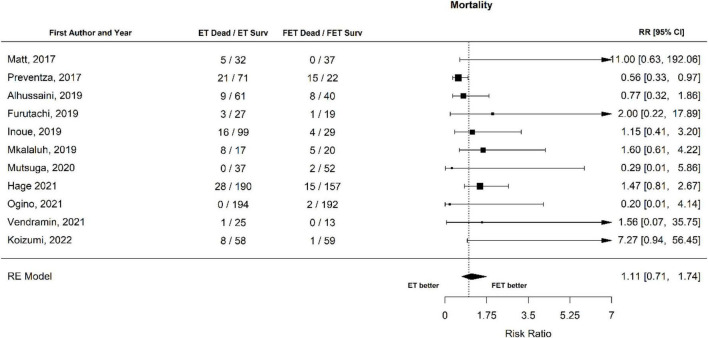
Relative risk of mortality in patients undergoing aortic interventions with conventional elephant trunk (ET) vs. frozen elephant trunk (FET). Data only from studies published between 2017 and 2021. Forest plot *p* = 0.646.

**FIGURE 11:**
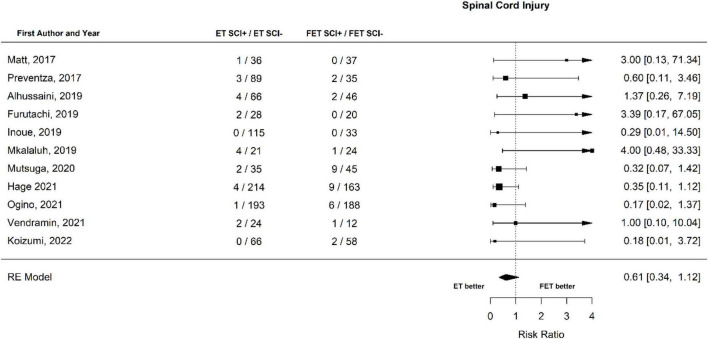
Relative risk of spinal cord injury (SCI) and hemiplegia/paraplegia in patients undergoing aortic interventions with conventional elephant trunk (ET) vs. frozen elephant trunk (FET). Data only from studies published between 2017 and 2021. Forest plot. ET vs. FET: *p* = 0.113.

We hypothesized that the evolution of techniques and the availability of newer devices, and the subsequent experience, particularly for FET, could have a positive impact on the outcomes and likely reduce mortality and perioperative complications. To test this hypothesis, the statistics were re-run including only the articles that were published between 2017 and 2021 and contained data from newer studies ranging up to 2021. The results of this second analysis are presented below and are shown in Table 3 and Figures 10, 11 and Supplementary Figures 1–8.

In the papers published in the last 5 years, the overall mortality was 99 out of 910 (10.9%) in patients that underwent ET and 53 out of 693 (7.6%) in patients that underwent FET. There was no statistically significant difference in the risk between ET and FET. The RR for mortality of patients that underwent ET vs. patients that underwent FET was 1.11 [0.71 to 1.74] (*p* = 0.65), (Forest plot, Figure 10). There was no effect of age or sex on this outcome (Table 3).

When spinal cord injury and related neurological implications were examined in the papers published between 2017 and 2021, the RR of SCI of patients that underwent ET vs. patients that underwent FET was 0.61 [0.34 to 1.12]. In contrast with what was observed when all papers were examined, the trend for lower SCI did not reach statistical significance (*p* = 0.112) (Forest plot, Figure 11). A non-significant trend for SCI was observed with increasing age (*p* = 0.131). Male sex was associated with higher odds for SCI (*p* = 0.029).

The RR for stroke in the papers published between 2017 and 2021 was 1.06 [0.76 to 1.47] (Table 3). Increasing age was found to increase the risk for stroke (*p* = 0.044). There was no significant effect of sex on this outcome (Table 3).

When renal failure was examined in papers published in the last 5 years, the RR of ET vs. FET was 1.15 [0.71 to 1.88]. There was no significant effect of either age or sex on the outcome of renal failure (Table 3).

## Discussion

The ET technique was introduced in the early 80 s for the correction of aortic arch aneurysms. The FET technique was introduced and developed between 1996 and 2003 in order to achieve similar results, to optimize the placement of the prosthesis in a single operative session and to combat the high interstage mortality that had been observed with the two-step ET technique (11, 13, 42). Since then, significant technical developments have occurred, including patient-tailored prostheses which are becoming increasingly popular (11).

The examined studies report conflicting results on the outcomes examined in this meta-analysis. Most of the examined studies reported no differences in mortality between ET and FET (21–23, 25–29, 31, 34–36, 38–41). Shrestha et al. (37) found significantly higher 30-day mortality in the ET group (24.7%) than in the FET group (12.2%), *p* = 0.011. Hage et al. (24) found a higher mortality rate after ET (13%) vs. FET (9%) (*p* = 0.022). According to a previous meta-analysis (12), the FET technique appears to have a lower perioperative mortality rate.

Our results also indicate that FET is probably associated with lower early mortality. As the RR (1.37 [1.04 to 1.81], *p* = 0.027) is relatively close to 1, this observation needs to be interpreted with caution. When papers published in the last 5 years (2017–2021) were examined, there was no statistically significant difference in early mortality. We hypothesized that the availability of better devices and the evolution of techniques and training of the personnel would contribute to a reduction of the mortality rate of FET. However, the mortality rate was similar in papers published in all years (7.9%) and in the last 5 years (7.6%). Notably, mortality in aortic arch replacement, especially type A dissection is confounded by several operative and patient specific factors, encumbering this comparison in a retrospective fashion.

Interestingly, while the FET technique has over the years a constant relatively low mortality rate, the ET technique has evolved and achieved reduced mortality rates in the last years. This improvement in mortality rate was observed in ET group within the last 5 years (10.9%) as compared to earlier years (14.8%). The improvement in mortality rates with primary procedure in conventional ET could be at least partially attributed to the evolution of the used techniques in cerebral protection and cardiopulmonary bypass.

It must be noted here, that this meta-analysis only examined early mortality up to 30 days after intervention. It is not known if the long-term mortality follows similar trends. In fact, the ET techniques is followed by a second procedure, which potentially increases the complication and mortality rate significantly. Nevertheless, the reported late mortality rates were conflicted and most studies report no differences after prolonged follow up. Jacob et al. (27) reported similar late mortality at follow-up (average follow-up ET 48 months, FET 23 months). Uchida et al. (39) found a survival rate of 69.0% after conventional arch replacement and 95.3% after FET after 5 years follow-up. Hofferberth et al. (25) reported survival rate 80% in ET vs. 87% in FET after a mean follow-up of 50 months. Mutsuga et al found an overall survival of 83% after ET and was 73% after FET at 5 years with no significant difference (*p* = 0.73). Koizumi et al. (28) reported no differences in mortality after 3 years follow-up.

According to previous studies there is a relatively high risk for a wide range of neurological complications in aortic arch surgery and these complications are well recorded (13, 43). However, there are studies showing that FET has more neurological complications when it comes to spinal cord injury, even suggesting that ET should be considered for patients who are expected to have more time under circulatory arrest in more moderate hypothermia. Leontyev et al. (29) reported lower occurrence of new-onset paraplegia after ET (4.0%) than after FET (21.7%), *p* < 0.001. Ogino et al reported lower rates of stroke (2.2 vs. 5.7% respectively, *p* = 0.022) and paraplegia (0 vs. 1.6%, *p* = 0.023) after ET as compared to FET respectively. These findings are partially confirmed by our analysis of all examined articles, where the RR for SCI of ET vs. FET was found to be 0.53 (*p* = 0.004). Nevertheless, when only the papers published within the last 5 years were examined, the RR was 0.61 and there was no statistical significance. It is possible that current evolution of the FET technique and improved devices have contributed to this outcome. Male sex was found to be associated with higher risk for SCI only in the analysis of the papers published in the last 5 years.

Renal failure resulting from hypoperfusion is another important complication related to aortic arch surgery. We found no significant difference in renal failure for both techniques, although comparing the most recent studies, a significant effect of increasing age in the occurrence of renal failure was observed.

## Limitations

There are some limitations in this study.

There were no randomized controlled studies in this meta-analysis. Most of the studies were retrospective cohorts which results in selection bias.

Different types of devices were used in the examined studies and different operators with varying levels of experience performed the surgeries in centers with different settings and experiences. It is reasonable to assume that the outcomes of the interventions, particularly FET, are affected by the types of the devices used and the above mentioned parameters.

Not all the included studies contained data about all the examined outcomes (i.e., mortality, SCI, stroke, renal failure).

Further, it is also important to note that not all patients had the exact same type of lesion.

## Conclusion

In conclusion, aortic arch surgery is a complex procedure where the outcome is related to a myriad of confounding factors including the surgical technique. In a retrospective fashion where the effect of inclusion bias is evident, we attempted to compare the evolution of outcomes of the commonly used ET and FET techniques. We found that the procedures show comparable results in complication and mortality rates, while the outcomes have improved in the most recent studies. Although there is a trend in higher rates of spinal cord injury in FET, for a complete comparison, the results of the second procedure in the ET technique should also be considered. Future studies should focus on prospective analysis of arch replacement techniques and consider less invasive hybrid procedures where debranching of aortic vessels are followed by stenting of the aneurysmatic arch.

## Data availability statement

The original contributions presented in this study are included in the article/Supplementary material, further inquiries can be directed to the corresponding author.

## Author contributions

AM: conceptualization and writing—original draft preparation. EB and EN: methodology. GP: software. SG: validation and writing—review and editing. OP: formal analysis and data curation. JR: investigation. JM: project administration. All authors have read and agreed to the published version of the manuscript.
