# Supplementary material for: The evolution of arch surgery: Frozen elephant trunk or conventional elephant trunk?
Source: Front Cardiovasc Med. 2022 Oct 20;9:999314. doi: 10.3389/fcvm.2022.999314 (PMC9630467; doi:10.3389/fcvm.2022.999314)
Supplement: Supplementary file 1 [file Data_Sheet_1.docx]

# Supplementary Material

The evolution of arch surgery: Frozen Elephant Trunk or Conventional Elephant Trunk?

Amalia I Moula, BSc, Jamie LR Romeo, MD PhD, Gianmarco Parise, PhD, Orlando Parise, MD, MSc, Jos G Maessen, MD, PhD, Ehsan Natour, MD, PhD, Elham Bidar, MD PhD, Sandro Gelsomino, MD, PhD.

Cardiovascular Center, University Hospital Maastricht, CARIM School for Cardiovascular Disease, University of Maastricht

**Supplement A**

**Search Queries:**

The following query was used in PubMed:

("Aortic Aneurysm, Thoracic"[Mesh] OR "Aorta, Thoracic"[Mesh] OR (aort*[Title/Abstract]) OR Arch[Title/Abstract] OR Dissection[Title/Abstract] OR Aneurysm[Title/Abstract] OR thora*[Title/Abstract] OR "Aneurysm, Dissecting"[Mesh]) AND (Elephant trunk[Title/Abstract] AND ((Frozen[Title/Abstract]) OR stented[Title/Abstract])) AND (("Treatment Outcome"[Mesh] OR outcome) OR ("Postoperative Complications"[Mesh] OR complications) OR ("Mortality"[Mesh] OR mortality) OR (“Morbidity” [Mesh] OR morbidity) OR ("Stroke"[Mesh] OR stroke OR “cerebral vascular accident” OR “cerebrovascular accident”) OR ("Spinal Cord Ischemia"[Mesh] OR “spinal cord ischemia”) OR ("Spinal Cord Injuries"[Mesh] OR “spinal cord injury”) OR ("Paraplegia"[Mesh] OR paraplegia) OR ("Paraparesis"[Mesh] OR paraparesis)).

The Web of Science query was: ALL=(("elephant trunk" AND "frozen" AND ("aorta" OR "aortic")) AND ("mortality" OR "spinal cord injury" OR "paraplegia" OR "stroke" OR "renal failure")).

The Cochrane library query was: "elephant trunk" in Title Abstract Keyword.

The Scopus query was: (TITLE-ABS-KEY (("elephant trunk" OR "elephant AND trunk" OR "thoracic surgery") AND frozen)) AND (TITLE-ABS-KEY(mortality OR stroke OR "spinal cord injury" OR "renal failure")) AND (LIMIT-TO (SUBJAREA , "MEDI")) AND (LIMIT-TO (DOCTYPE , "ar")) AND (LIMIT-TO (LANGUAGE, "English).

**Supplement B**

**SUPPLEMENTAL TABLE AND FIGURES:**

|  | **Question** | Mean | SD |
| --- | --- | --- | --- |
| 1 | Study hypothesis/aim/objective described? | 1.00 | 0.00 |
| 2 | Main outcomes described in the introduction or methods? | 0.77 | 0.43 |
| 3 | Participant characteristics described? | 0.95 | 0.21 |
| 4 | Contacted participants representative? | 0.82 | 0.39 |
| 5 | Prepared participants representative? | 0.91 | 0.29 |
| 6 | Participants recruited from the same population? | 0.86 | 0.35 |
| 7 | Participants recruited over the same time? | 0.68 | 0.48 |
| 8 | Measures and experimental tasks described? | 1.00 | 0.00 |
| 9 | Main outcome measures valid and reliable? | 1.00 | 0.00 |
| 10 | Task engagement assessed? | 1.00 | 0.00 |
| 11 | Confounders described and controlled for? | 0.36 | 0.49 |
| 12 | Statistical tests appropriate? | 1.00 | 0.00 |
| 13 | Main findings described? | 1.00 | 0.00 |
| 14 | Estimates of the random variability in data main outcomes? | 1.00 | 0.00 |
| 15 | Probability values reported? | 1.00 | 0.00 |
| 16 | Withdrawals and drop‐outs reported? | 0.77 | 0.43 |
| 17 | Data dredging made clear? | 0.73 | 0.46 |
| 18 | Sufficient power analysis provided? | 0.00 | 0.00 |

**Supplemental Table 1**. Quality assessment of the included articles. All items have a maximum score of 1.00 except for item 11, which has a maximum score of 2.00.


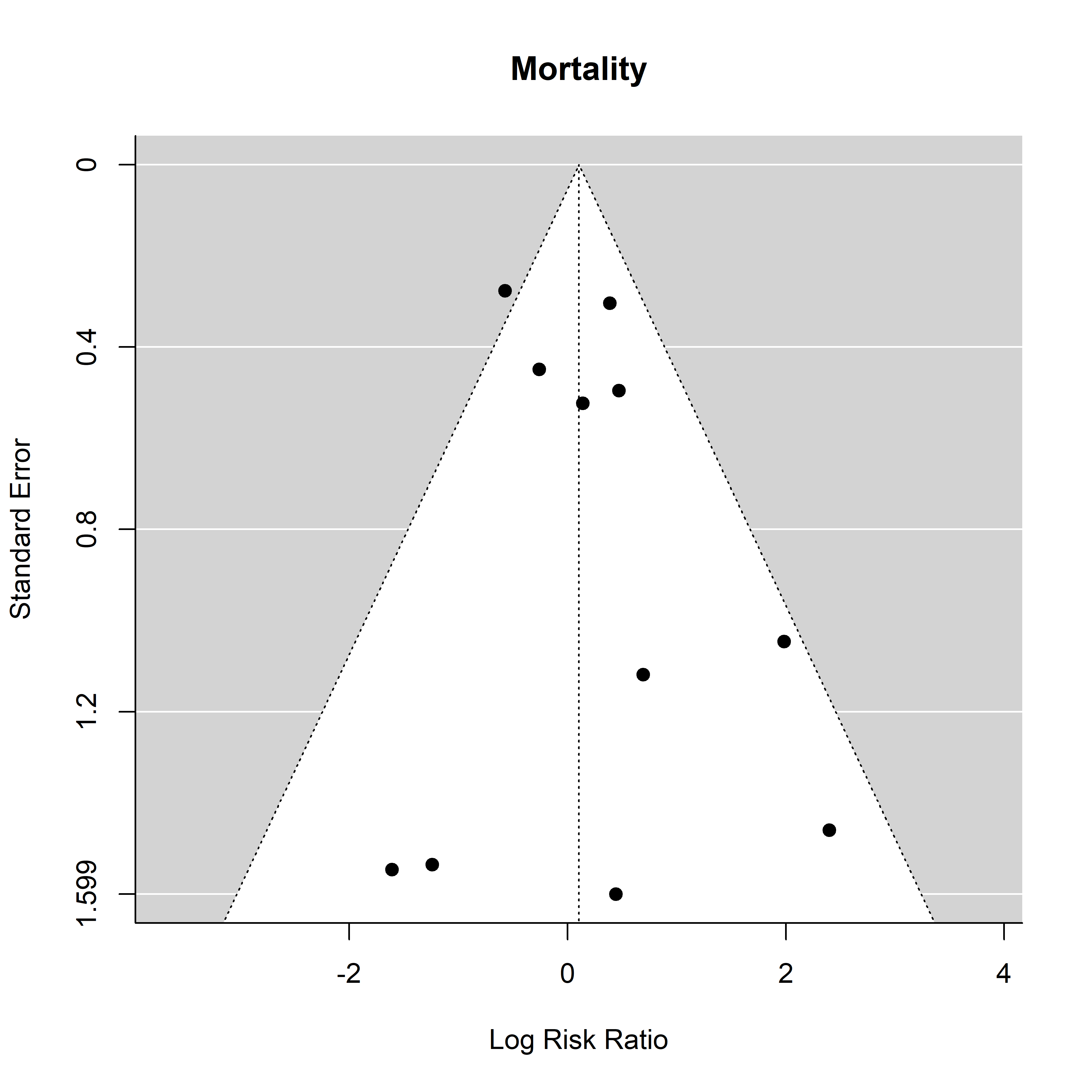


**Supplemental Figure 1:** Relative risk of mortality in patients undergoing aortic interventions with conventional elephant trunk (ET) versus frozen elephant trunk (FET). Data only from studies published between 2017 and 2021. Funnel plot.


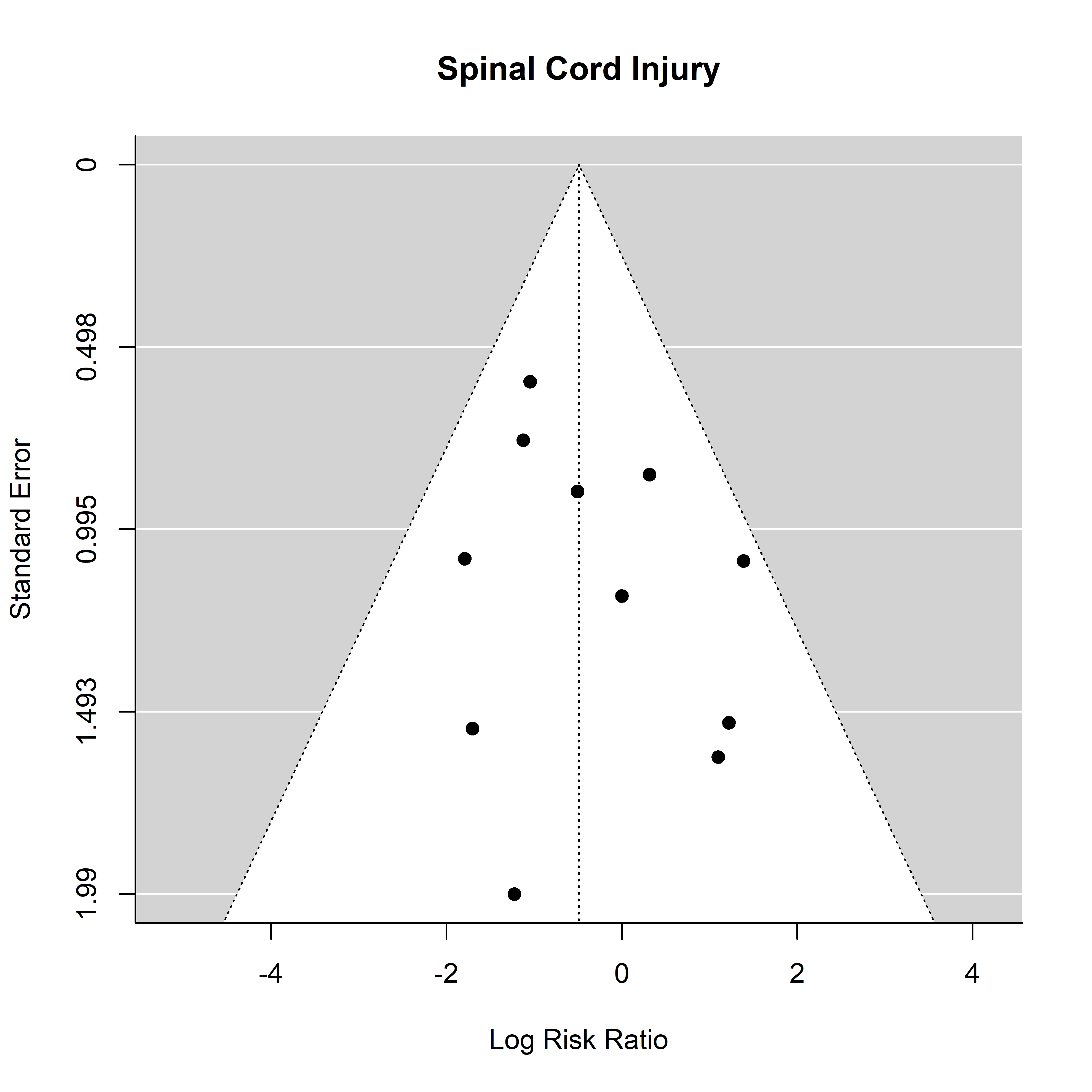


**Supplemental Figure 2:** Relative risk of spinal cord injury (SCI) and hemiplegia/paraplegia in patients undergoing aortic interventions with conventional elephant trunk (ET) versus frozen elephant trunk (FET). Data only from studies published between 2017 and 2021. Funnel plot.


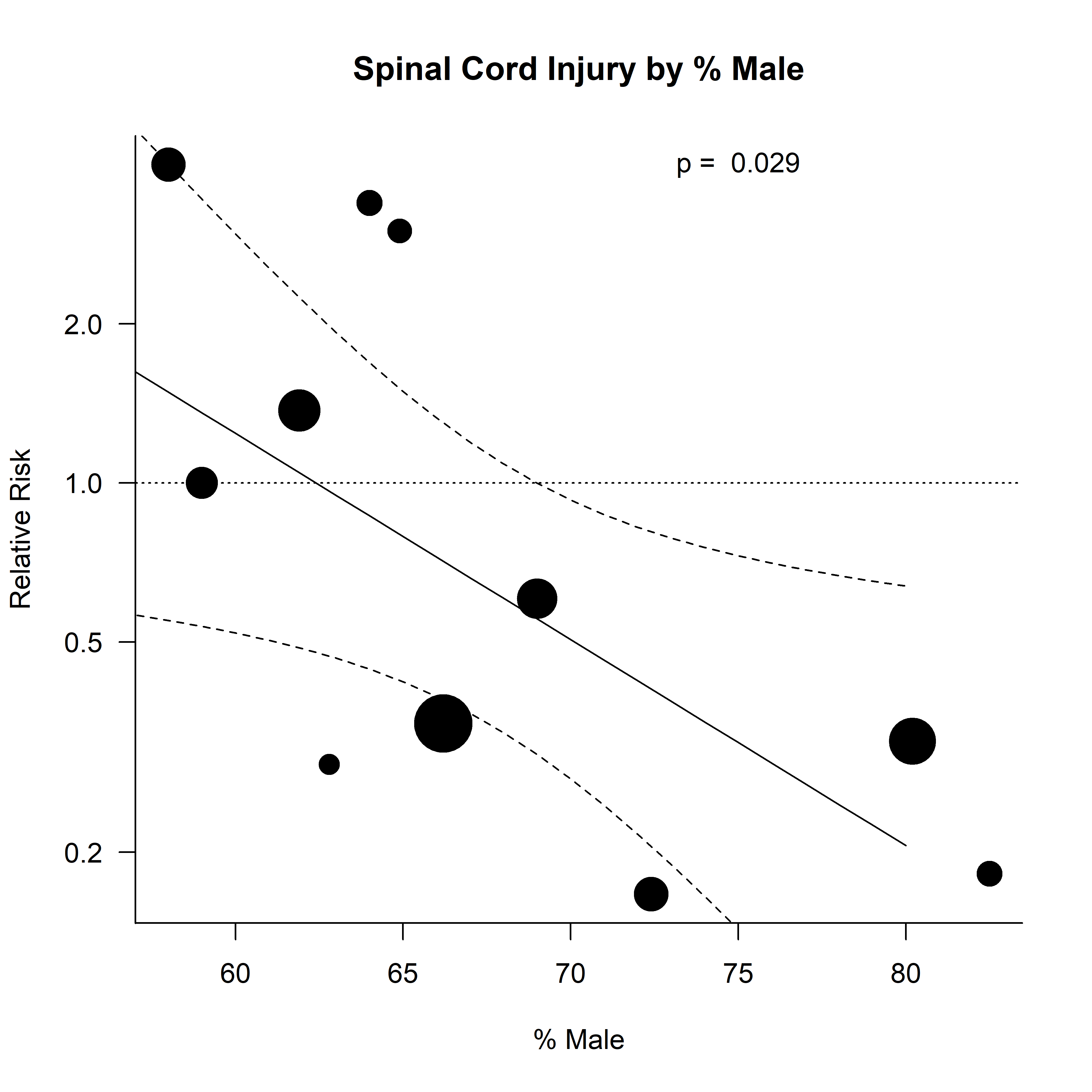


**Supplemental Figure 3**: Effect of sex (%male) on the relative risk of spinal cord injury (SCI) and hemiplegia/paraplegia in patients undergoing arterial intervention with conventional elephant trunk (ET) and frozen elephant trunk (FET). Data only from studies published between 2017 and 2021.

**
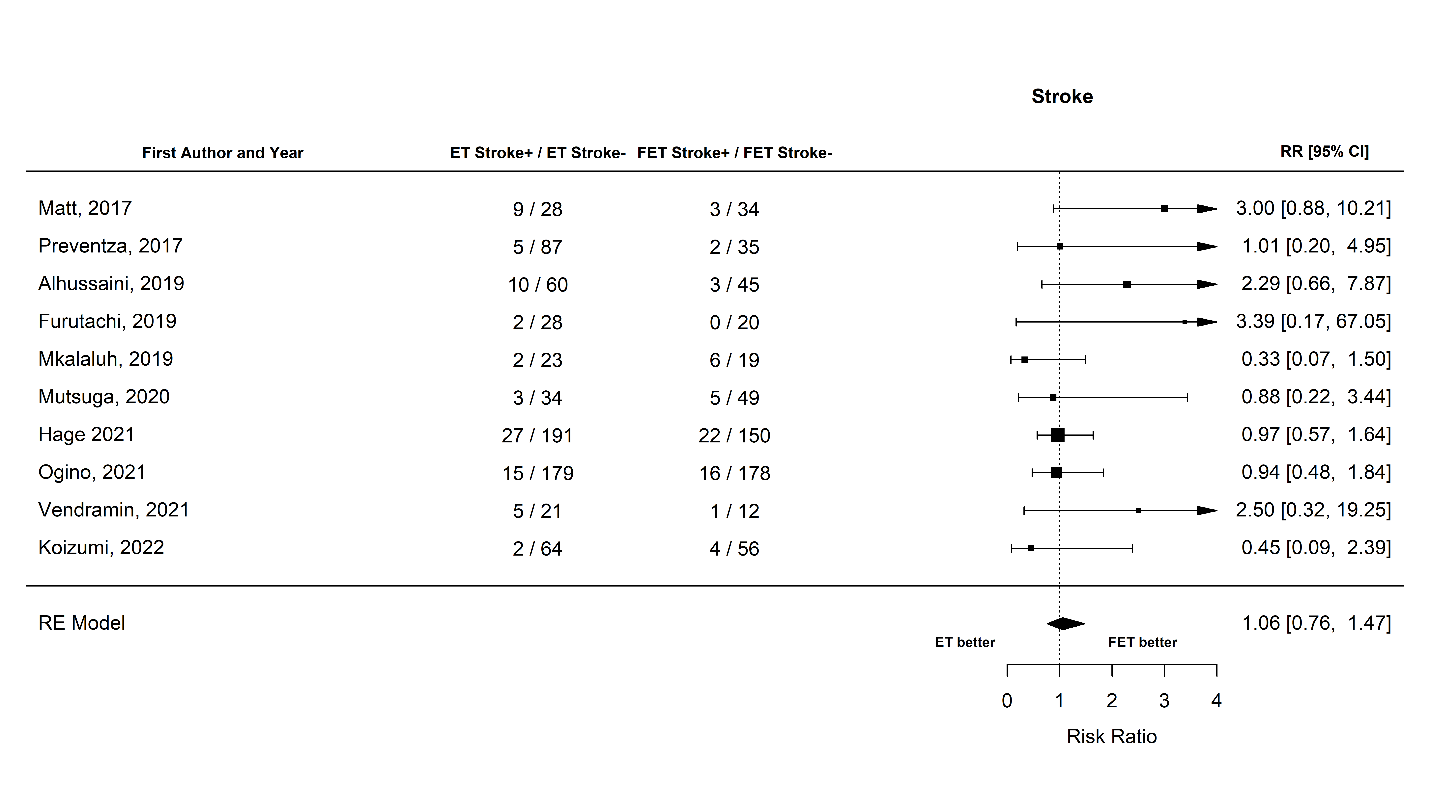
**

**Supplemental Figure 4:** Relative risk of stroke in patients undergoing aortic interventions with conventional elephant trunk (ET) versus frozen elephant trunk (FET). Data only from studies published between 2017 and 2021. Forest plot.


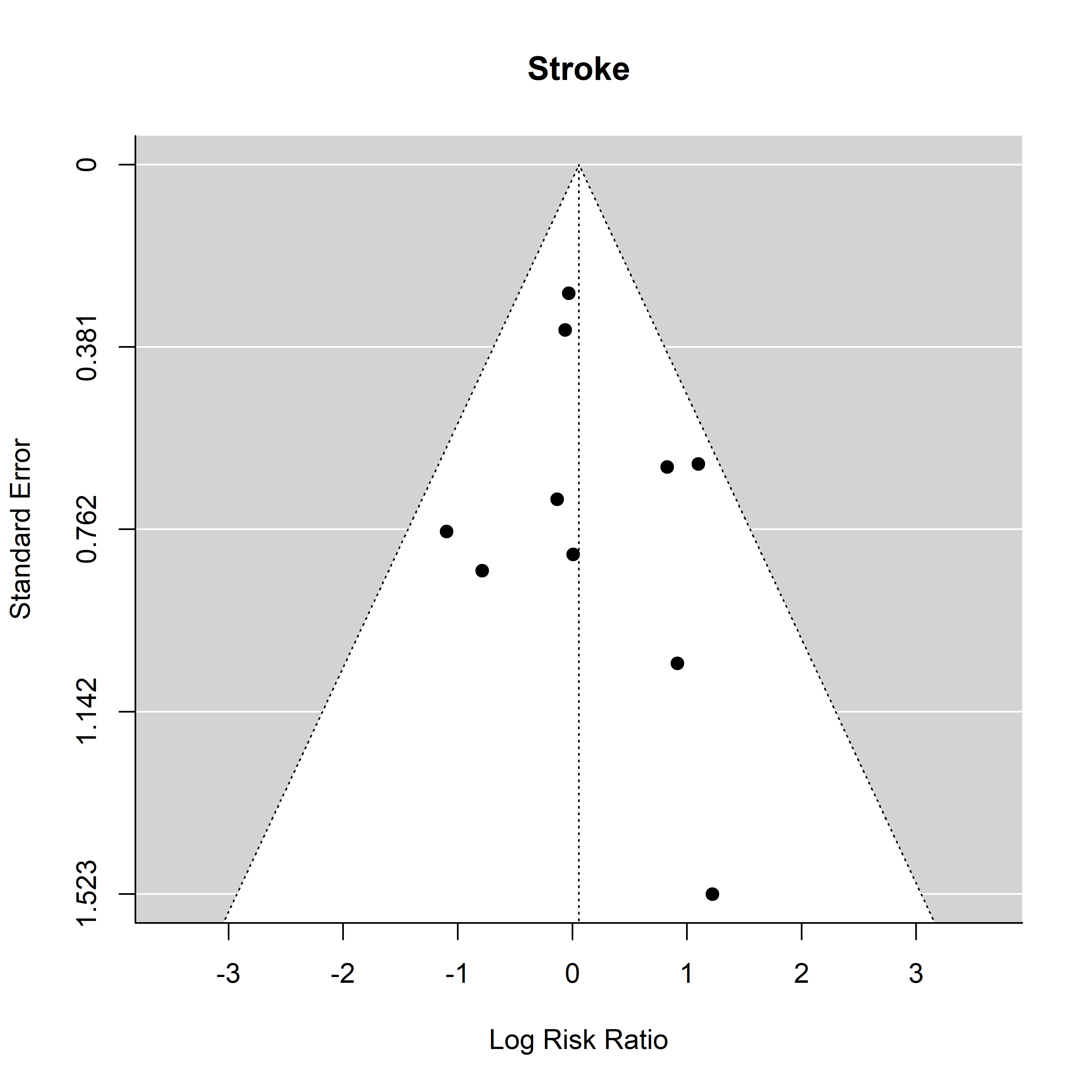


**Supplemental Figure 5:** Relative risk of stroke in patients undergoing aortic interventions with conventional elephant trunk (ET) versus frozen elephant trunk (FET). Data only from studies published between 2017 and 2021. Funnel plot.


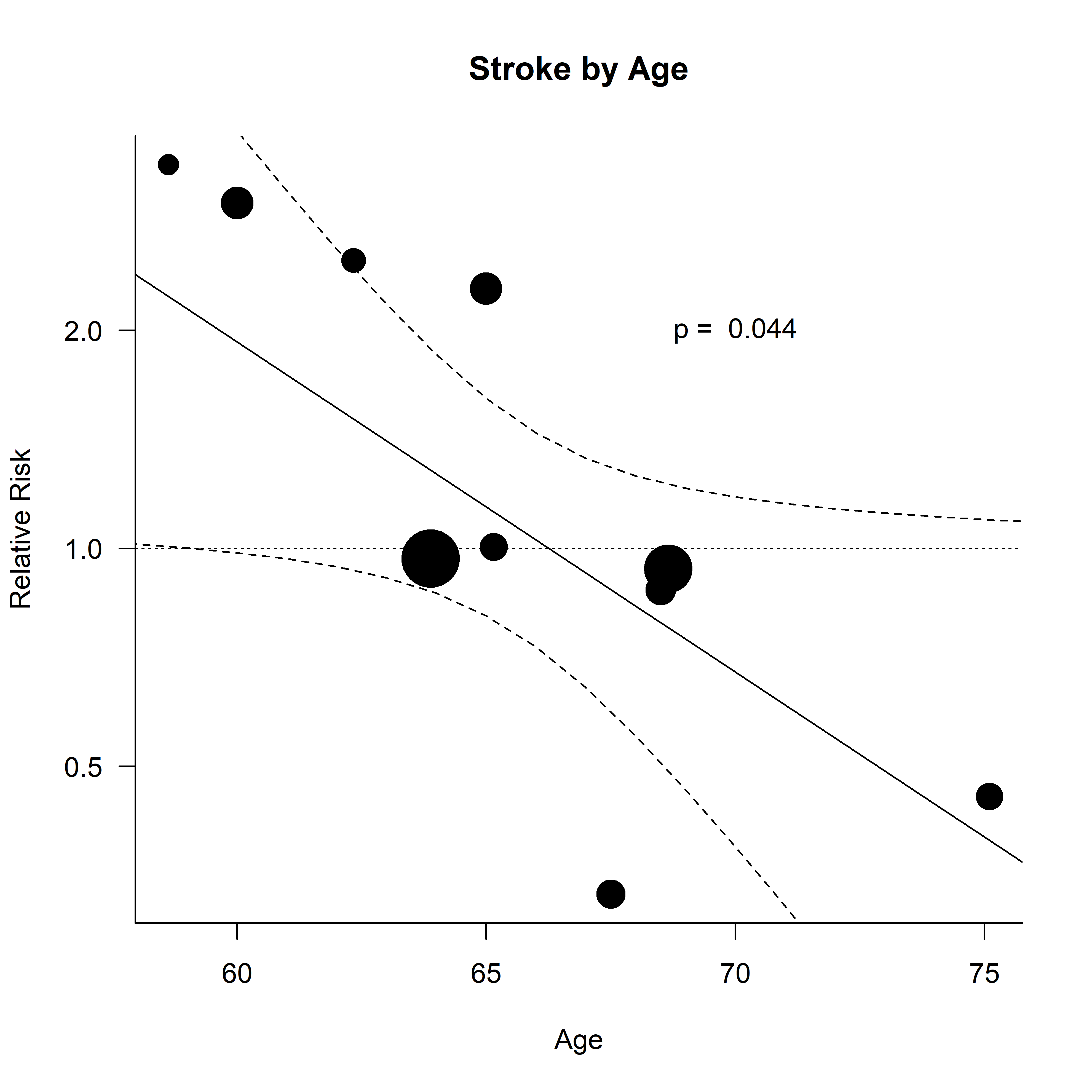


**Supplemental Figure 6**: Effect of age on the relative risk of stroke in patients undergoing arterial intervention with conventional elephant trunk (ET) and frozen elephant trunk (FET). Data only from studies published between 2017 and 2021.


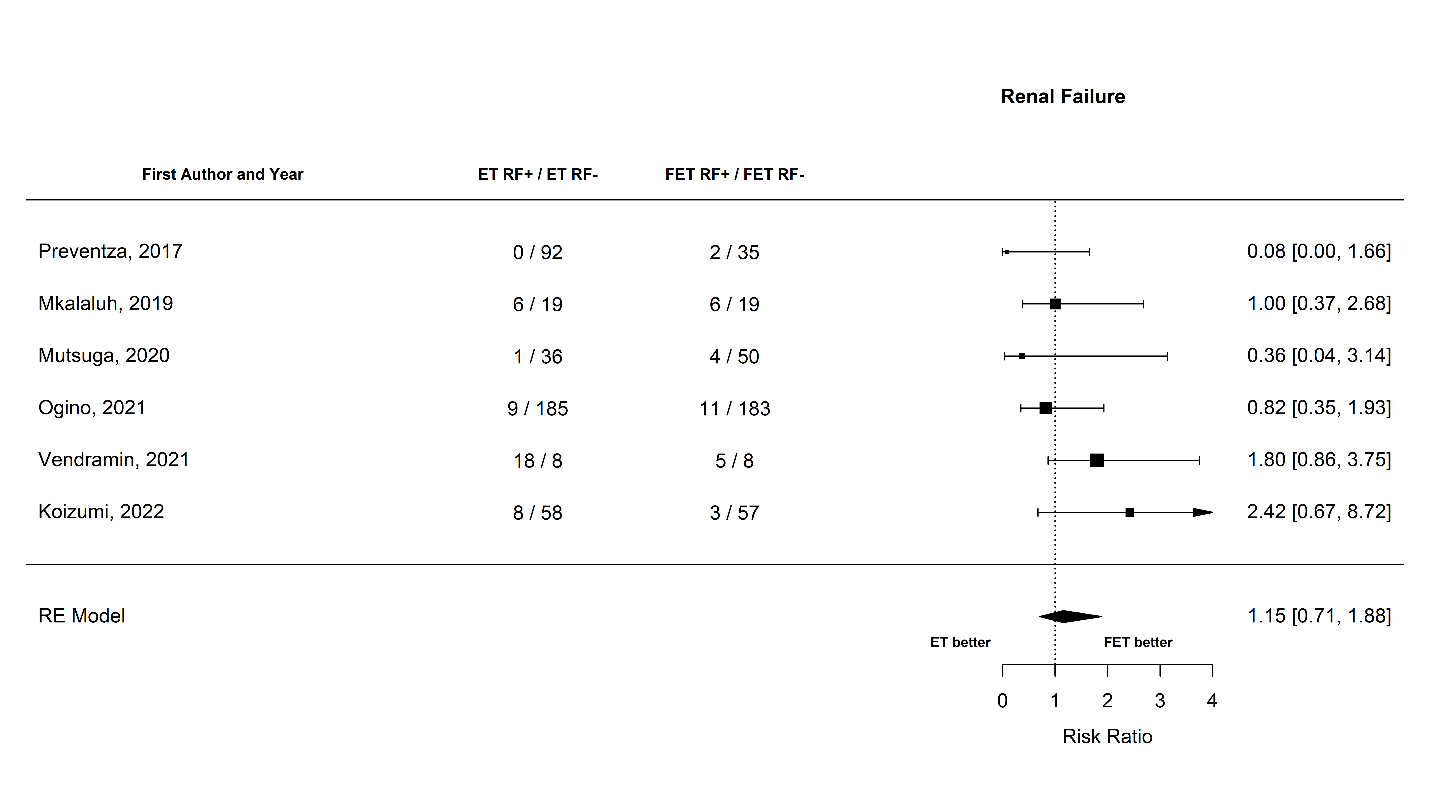


**Supplemental Figure 7:** Relative risk of renal failure (RF) in patients undergoing aortic interventions with conventional elephant trunk (ET) versus frozen elephant trunk (FET). Data only from studies published between 2017 and 2021. Forest plot.


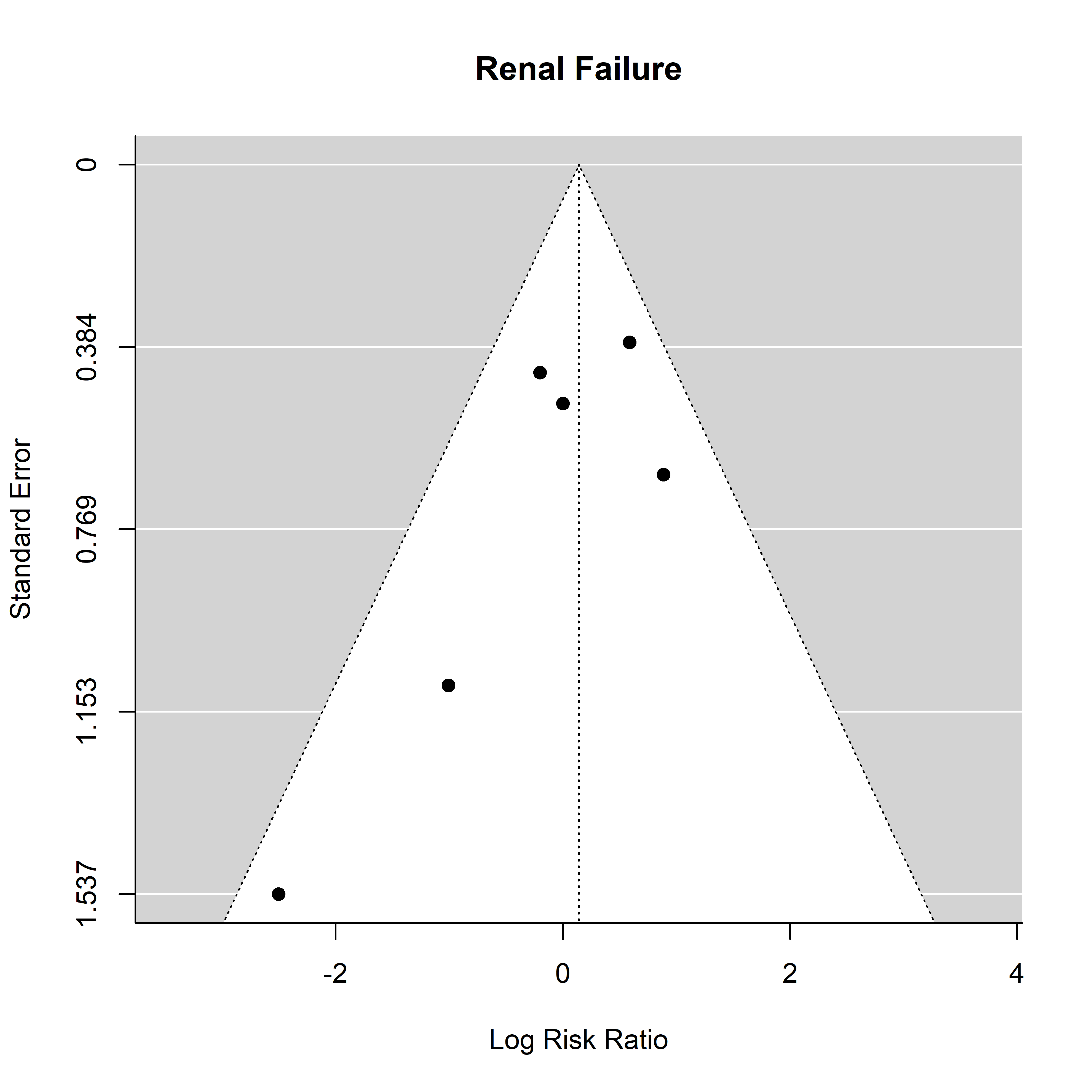


**Supplemental Figure 8:** Relative risk of renal failure (RF) in patients undergoing aortic interventions with conventional elephant trunk (ET) versus frozen elephant trunk (FET). Data only from studies published between 2017 and 2021. Funnel plot.
